# Long Term Follow-Up of Patients with Systemic Right Ventricle and Biventricular Physiology: A Single Centre Experience

**DOI:** 10.3390/jcdd10050219

**Published:** 2023-05-17

**Authors:** Cristina Ciuca, Anna Balducci, Emanuela Angeli, Mariateresa Di Dio, Gabriele Egidy Assenza, Elisabetta Mariucci, Luca Ragni, Luigi Lovato, Fabio Niro, Valentina Gesuete, Lucio Careddu, Ylenia Bartolacelli, Ambra Bulgarelli, Andrea Donti, Gaetano Domenico Gargiulo

**Affiliations:** 1 Pediatric Cardiology and Adult Congenital Heart Disease Program, Department of Cardio-Thoracic and Vascular Medicine, IRCCS Azienda Ospedaliero-Universitaria di Bologna, 40138 Bologna, Italy; 2 Pediatric Cardiac Surgery and Adult Congenital Heart Disease, Department of Cardio-Thoracic and Vascular Medicine, IRCCS Azienda Ospedaliero-Universitaria di Bologna, 40138 Bologna, Italy; 3 Radiology Unit, Department of Cardio-Thoracic and Vascular Medicine, IRCCS Azienda Ospedaliero-Universitaria di Bologna, 40138 Bologna, Italy

**Keywords:** systemic right ventricle, adults with congenital heart disease, atrial switch operation, congenitally corrected transposition of the great arteries, Mustard operation, Senning operation

## Abstract

Background: A progressively increasing prevalence of congenital heart disease (CHD) in adulthood has been noticed in recent decades; CHD cases with a systemic right ventricle have a poorer outcome. Methods: Seventy-three patients with SRV evaluated in an outpatient clinic between 2014 and 2020 were enrolled in this study. Thirty-four patients had a transposition of the great arteries treated with an atrial switch operation; 39 patients had a congenitally corrected transposition of the great arteries (ccTGA). Results: Mean age at the first evaluation was 29.6 ± 14.2 years; 48% of the patients were female. The NYHA class at the visit was III or IV in 14% of the cases. Thirteen patients had at least one previous pregnancy. In 25% of the cases, complications occurred during pregnancy. Survival free from adverse events was 98.6% at one year and 90% at 6-year follow-up without any difference between the two groups. Two patients died and one received heart transplantation during follow-up. The most common adverse event during follow-up was the presence of arrhythmia requiring hospitalization (27.1%), followed by heart failure (12.3%). The presence of LGE together with lower exercise capacity, higher NYHA class and more dilated and/or hypokinetic RV predicted a poorer outcome. Quality of life was similar to the QoL of the Italian population. Conclusions: Long-term follow-up of patients with a systemic right ventricle is characterized by a high incidence of clinical events, prevalently arrhythmias and heart failure, which cause most of the unscheduled hospitalizations.

## 1. Introduction

A progressively increasing prevalence of adults with congenital heart disease (ACHD) was noticed in recent decades and estimated to be 0.6% in 2010. (1) Almost 12% of patients with CHD have a right ventricle that sustains the systemic circulation (systemic right ventricle, SRV). The CHDs associated with an SRV and biventricular circulation are congenitally corrected transposition of the great arteries (ccTGA) and transposition of the great arteries (TGA) treated with an atrial switch operation (AS). The ccTGA is a rare form of CHD, characterized by atrioventricular and ventriculoarterial discordance, with a prevalence of 3 in over 100,000 live births. Transposition of the great arteries (TGA) is a frequent cyanotic CHD, with an incidence ranging from 20.1 to 30.5 in 100,000 live births and characterized by atrioventricular concordance and ventriculoarterial discordance. A significant improvement in survival was achieved after the introduction in 1957 of the atrial switch operation (AS) by Ake Senning [1], subsequently modified by William Mustard in 1963 [2]. Since the 1990s, the choice therapy for TGA has been the arterial switch operation, which is associated with a lower complication rate and better long-term outcome. SRV is associated with a high risk of adverse events, prevalently arrythmias and heart failure [3,4]. Severe tricuspid regurgitation, reduced exercise capacity, chronotropic incompetence, and the presence of fibrosis have been previously described as predictors of a poorer outcome [5,6,7,8]. The aims of this study were to evaluate long-term outcomes in patients with a systemic right ventricle and to identify predictors of a poorer outcome.

## 2. Methods

This study was designed as a retrospective-prospective analysis of SRV in a referral congenital heart disease center (CHDC). All patients with a previous Senning/Mustard operation or ccTGA evaluated in the outpatient clinic for a routine clinical follow-up planned between 2014 and September 2021 were enrolled in this study. All patients participated in at least two planned visits during the study period. All clinical events were recorded.

Patients were evaluated using routine clinical practice with clinical examination, ECG, HOLTER ECG, echocardiography, cardiopulmonary exercise test (CPET), and cardiac MRI. Adverse clinical events included death, heart transplantation, active presence on the waiting list for HT, heart failure requiring hospitalization, stroke, myocardial infarction, and arrythmias requiring hospitalization. During follow-up, survival free from heart transplantation and survival free from adverse events were evaluated. A poorer outcome was defined as the presence of adverse clinical events during the study.

Echocardiography was performed using the European Association of Cardiovascular Imaging/American Society of Echocardiography guidelines and recommendations [9,10].

Cardiopulmonary exercise tests (CPETs) were performed on an upright cycle ergometer (Cardioline, Italy) using a continuous ramp protocol (10 watt/min) until muscular exhaustion, with continuous monitoring of expiratory gas. 

Cardiovascular magnetic resonance (CMR) was performed using a 1.5-Tesla Magnetic Resonance Unit (Philips Medica System, The Netherlands). The presence of late gadolinium enhancement (LGE) was evaluated using a segmented fast low-angle shot inversion recovery sequence 15 min after injection of 0.15 mmol/kg IV gadolinium-DPTA. A standardized CMR protocol for assessment status post Mustard/Senning operation was performed. A short-axis contiguous stack of 7-mm cine images (3-mm gap) was acquired for quantification of ventricular function and mass using Simpson’s method.

The Short Form-36, derived from the General Health Survey of the Medical Outcomes Study by Stewart and colleague, is one of the most widely used generic measures of health-related quality of life and has been shown to discriminate between subjects with different chronic conditions and between subjects with different severity levels of the same disease [11]. The SF-36 is a generic multi-item questionnaire comprising 36 questions in eight domains: physical functioning, role limitations due to physical health, bodily pain, general health perceptions, vitality, social functioning, role limitations due to emotional problems, and mental health, with scores ranging from 0 to 100, where higher scores represent better quality of life. The results were compared with those of the Italian population [12].

### Statistical Analysis 

The statistical analysis was performed with the Statistical Package for Social Science (SPSS) 21.0. Continuous variables are reported as mean ± SD and categorical variables as frequencies and percentages. Differences between groups were assessed using the two-tailed Student’s *t*-test for continuous variables and the chi-square test or Fisher exact test for the categorical variables. The cumulative incidences of clinical events at follow-up were assessed with the Kaplan-Meier method, and the log-rank test was used for comparison between groups. Cox regression was performed to identify predictors of mortality or HT. All variables with a *p* ≤ 0.05 were included in the multivariable analysis. A *p* value < 0.05 was considered statistically significant.

## 3. Results 

Overall, 73 patients with a systemic right ventricle (SRV) were included in this study. The etiology of SRV was 47% atrial switch (22% previous Mustard operation and 25% previous Senning operation) and 53% congenitally corrected transposition of the great arteries. Mean follow-up timeframe was 8 ± 1 years.

Mean age at the first evaluation was 29.6 ± 9 years with no difference between groups (31.4 ± 7.1 in the AS group and 27.8 ± 8.1 in the ccTGA group, *p* = 0.283). Thirty-five patients (47.9%) were female. Overall, 86% of patients had a good exercise capacity with functional NYHA class I/II with no significative differences between groups, while 14% of patients had an advanced NYHA class. 

Fifty patients underwent at least one surgical intervention: all patients in the AS group and 17 patients (43.5%) with ccTGA. Indications for surgery in the ccTGA group were prevalently the associated lesions: pulmonary outflow obstruction (58.8%), VSD (41.2%), coarctation of the aorta (11.7%), ASD (41%), and severe TR (29.4%). Twelve patients (16.4%) underwent a second cardiac surgery [in the AS group prevalently for baffle obstruction or leak (three patients) or systemic AV valve replacement (two patients); in the ccTGA group, the indications for a second surgery were tricuspid valve/prothesis replacement, VSD closure, and pulmonary artery reconstruction in previous pulmonary bending for large VSD], and four patients (5.4%) underwent a third cardiac surgery (two in the AS group and two in the ccTGA group). 

At the first evaluation, nine patients (12.3%) presented with either atrial fibrillation or atrial flutter, with a significant difference between the two groups: 17.6% in the AS group versus 7.6% in the ccTGA group (*p* = 0.02). Moreover, 15 patients (20.5%) had a permanent pacemaker (PM): nine patients with a ccTGA (23%) and six patients (17.6%) with AS. Furthermore, five patients had an ICD (2 in the AS group and 3 in the ccTGA group), and in one Senning patient, an upgrade of the PM to CRT-P was performed. Mean age of PM/ICD implantation was 30 ± 16 years old. 

Echocardiography data are presented in Table 1. Most patients presented with dilated right ventricle (RV), overall, the echo end-diastolic and end-systolic areas were 20.1 ± 5.3 cm^2^/m^2^ and 12.9 ± 4.4 cm^2^/m^2^, respectively. The SRV of AS patients was significantly more dilated. Basal and mid-RV diameters were also increased, while the longitudinal diameter was normal. Overall, systolic function was normal or slightly impaired: FAC 36.8 ± 10.3%, TAPSE 15.3 ± 3.8 mm, and S wave TDI 8.7 ± 2.3 cm/s. Free wall and six segments LS values were −12.1 ± −5.1% and, −10.9 ± −4.5%, respectively. Systemic tricuspid regurgitation (sTR) was common: 23.2% of patients had moderate sTR, 17.8% had moderate-to-severe sTR, and 8.2% had severe sTR (no difference between groups). Ten patients already had a mechanical tricuspid prosthesis (eight patients with ccTGA, *p* < 0.001). The left atrium was often dilated: volume 39 ± 25 mL/m^2^, area 12.3 ± 4.7 cm^2^/m^2^, with no difference between groups. Stenosis at the pulmonary outflow was present in four patients. Baffle stenosis was present in six patients. 

Cardiac magnetic resonance was available for 52 patients (Table 2). Overall, a mild dilation and systolic impairment was noticed with larger volumes and lower ejection fractions for AS patients (EDV RV 127 ± 46 mL/m^2^ in the AS group versus 106 ± 30 mL/m^2^ in the CCTGA group, *p* = 0.07; RV EF 47 ± 11% in the AS group and 53 ± 12% in the ccTGA group, *p* = 0.06). The left ventricle had normal volumes and EF, with no differences between groups. Fibrosis was present in 42.3% of cases, with no significant differences between groups. Interestingly, the mean age of patients with LGE was 34 years, while the mean age of patients without evidence of LGE was 27 years (*p* = 0.01).

The cardiopulmonary exercise test was available for 57 patients. Data are presented in Table 3. Overall, a moderate impairment of exercise capacity was evidenced: peak VO2 24.5 ± 9.4 mL/kg/min = 67 ± 20 % of the predicted value, with a significantly lower performance for the patients with a previous atrial switch (22.2 ± 7.2 versus 27.2 ± 11, *p* = 0.04). 

**Figure 1:**
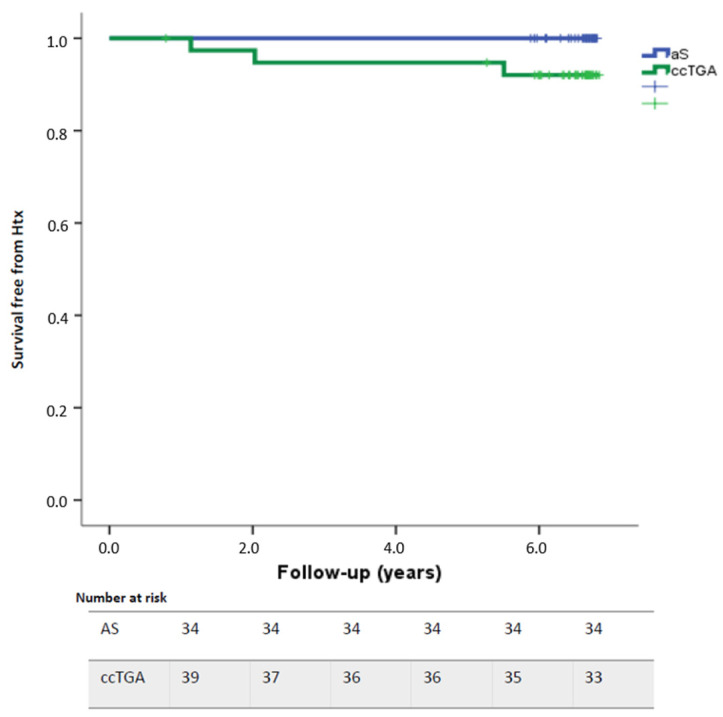
Survival free from adverse events during the study: Overall, one-year survival free from adverse events was 98.6% while 6-year survival free from adverse events was 90%.

Overall survival free from adverse events was 98.6% at one year and 90% at 6-year follow-up without difference between the two groups (*p* = 0.09) (Figure 1). Two patients died during follow-up: a 35-year-old man with ccTGA, VSD, ASD, pulmonary outflow stenosis, and coarctation of the aorta previously treated with de-coarctation and subsequently VSD, ASD closure, and treatment of sub-pulmonary stenosis in tricuspid valve replacement with a Carbomedics 25-mm mechanical prosthesis. The patient had a PM for complete AV block and a permanent atypical atrial flutter; he presented with a severe dilatation and dysfunction of the SRV. He was admitted for end-stage heart failure complicated by ventricular arrythmias and underwent ventricular assistance device implantation (VAD) during hospitalization as a bridge to transplantation but died because of multiorgan failure one month after the VAD implantation. The second patient was a 51-year-old woman with end-stage HF affected by ccTGA and a tricuspid mechanical prosthesis with severe SRV dysfunction, severe pulmonary hypertension, and severe restrictive pulmonary disease not eligible for HT.

Moreover, a 52-year-old patient underwent heart transplantation during follow-up. He had a ccTGA with severe SRV dysfunction. 

The most common adverse event during follow-up was the presence of arrhythmia requiring hospitalization (18 patients = 24.6%) followed by signs of heart failure requiring hospitalization (11 patients = 15%). One patient had a stroke during follow-up due to non-optimal adherence to anticoagulation therapy (a 55-year-old woman with a ccTGA and a mechanical tricuspid valve with a low INR value of 1.7). 

Arrythmia cases presented during follow-up included 41% atrial fibrillation, 39% atypical atrial flutter, and 20% IART. Three patients underwent transcatheter ablation for atypical atrial flutter.

Thirteen patients, four with a previous AS and nine with ccTGA (37.1% of the female population), had at least one previous pregnancy (range 1–4). Only in one case did the pregnancy follow an in vitro fertilization (IVF). In five cases, complications occurred during pregnancy (25%): two miscarriage, two cases of heart failure (the pregnancies were followed, and the fetuses had no complications), one case where eclampsia was followed by massive hemorrhage with necessity of hysterectomy (IVF pregnancy). Moreover, in one case, vertical transmission of ccTGA was evidenced, while in another case, dextrocardia was present in the newborn without other associated cardiac defects. 

Medical therapy was also registered. Almost one third of patients received diuretics, and more than 40% received beta-blockers or ACE-I/ARBs. A total of 27.1% of patients received oral anticoagulation therapy: 10 patients had a mechanical tricuspid prosthesis and 10 had atrial arrythmias requiring anticoagulation; except for three patients with atrial arrythmias treated with a new oral anticoagulant therapy, all other patients received warfarin. 

A univariate analysis was performed in order to identify the predictors of poorer outcome, which include a lower exercise capacity defined either as a higher NYHA class or a lower VO2 at CPET, a more dilated and dysfunctional SRV evaluated both with echocardiography and CMR, a higher degree of TR, and the presence of fibrosis documented at CMR. (Table 4)

**Figure 2:**
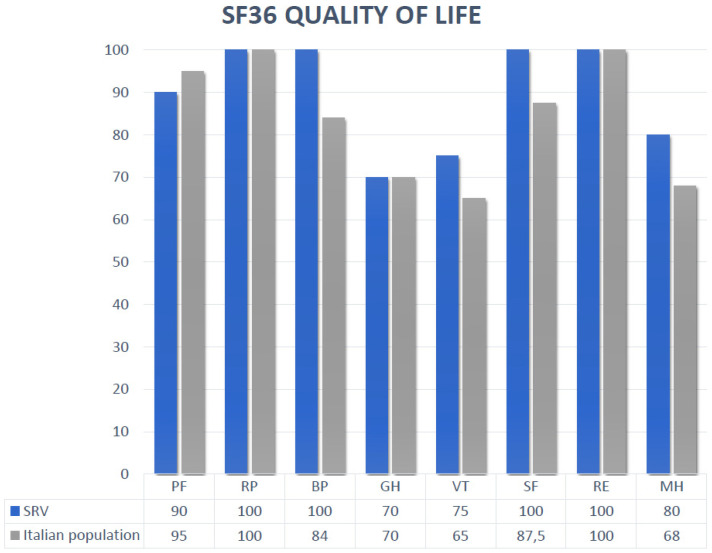
Quality of life evaluation: The SF-36 is a generic multi-item questionnaire comprising 36 questions in eight domains: physical functioning (PF) 83.8 ± 19.8; role physical (RP) 89.2 ± 29.9; bodily pain (BP) 90.3 ± 21.1, general health (GH) 67.8 ± 22, vitality (VT) 74 ± 16, social functioning (SF) 86.2 ± 17.8, role emotional (RE) 92.3 ± 25.5, and mental health (MH) 78.7 ± 15.9.

Quality of life, evaluated with the SF-36 test, was similar between groups and was indistinguishable from the QoL of the Italian population. Eight domains were evaluated, and the results were as follows: physical functioning (PF) 83.8 ± 19.8; role physical (RP) 89.2 ± 29.9; bodily pain (BP) 90.3 ± 21.1, general health (GH) 67.8 ± 22, vitality (VT) 74 ± 16, social functioning (SF) 86.2 ± 17.8, role emotional (RE) 92.3 ± 25.5, and mental health (MH) 78.7 ± 15.9. (Figure 2).

## 4. Discussion

Congenital heart diseases with a systemic right ventricle are a challenging situation for clinicians and need a multidisciplinary approach and a systematic follow-up to guarantee optimal management. Clinical evaluation should assess the NYHA class, the physiological state, and the coexistence of signs of heart failure, persistent or paroxysmal arrythmias, or other symptoms such as syncope, angina, or palpitation.

In recent decades, the treatment of heart failure associated with acquired cardiac disease changed dramatically with considerable improvement in survival and quality of life [13]. However, very few studies were conducted in ACHD patients with SRV, and the results are contrasting. 

Our study confirms a high incidence of adverse events during follow-up. The most common are supraventricular arrythmias requiring hospitalization (27.1%), supporting data presented in previous studies by Cuypers et al. (28%) [14]. Almost one third of patients received oral anticoagulant therapy (OAC); the main indications were mechanical prosthesis or atrial arrhythmias. However, non-vitamin K antagonist oral anticoagulants (NOACs) were used in a relatively low percentage of patients with atrial arrythmias. Further studies or trials are needed to verify long-term safety and efficacy of NOACs in these patients. 

The second cause of hospitalization was the worsening of heart failure (12.3%), similar to the data presented by Cuypers, where 14% of patients developed heart failure during the last decade of follow-up [14]. Optimization of medical therapy is crucial in these patients, but it needs evidenced-based trials. Therefore, interesting conclusions can be drawn from trials using dapagliflozin, such as in DAPA-HF [15,16], or sacubitril/valsartan, as in the Paradigm-HF trial [17] and further trials including these drugs could be performed in patients with SRV in order to assess efficacy and impact on outcome. 

Young adults should be empowered concerning their clinical condition, and therapeutic/follow-up strategies should be shared with them in order to improve compliance. Clinical evaluation should be performed every year or more frequently if necessary. Given the frequency of arrhythmic events, regular follow-ups with HOLTER ECG should be performed, and, in selected cases, longer ECG monitoring could be considered.

Echocardiographic evaluation is crucial and should be performed as the clinical evaluation every year; together with the standard evaluation including RV diameters, areas, and systolic function, the longitudinal function of the SRV evaluation should be routinely performed. 

CMR remains the gold standard to evaluate SRV because it better characterizes the myocardium, allowing the study and quantification of fibrosis. A progressive dilatation of the right ventricle was observed, with documented myocardial fibrosis in 42% of patients. In previous studies, extended fibrosis was documented during the autoptic heart examination [18]. Moreover, Rydman et al. demonstrated that LGE was present in half of patients and was associated with poorer outcome. The same results were seen in our cohort, with a crucial predictive role of the presence of LGE HR 12, 95% CI 2.6–59, *p* = 0.009 [7]. As demonstrated by Giardini et al., fibrosis is detected more frequently in older patients; in our study, the mean age of patients with LGE was 34 years, while the mean age of patients without evidence of LGE was 27 years [6].

In our study, RV dimensions and function, independently if evaluated with echocardiography or CMR, were strongly associated with poorer outcome. Even if RV systolic function might be difficult to evaluate with 2D echocardiography in these patients with previous surgical intervention, non-exceptional dextrocardia or mesocardia, we think, considering the prognostic value, that it should be routinely assessed during outpatient clinic evaluation, and change should be promptly confirmed by CMR or cardiac tomography when CMR is not feasible. Different cut-off values have been proposed by Irvitt et al. [19] for the evaluation of SRV. Moreover, an impairment in SVR strain values was observed even in the presence of normal systolic function, and it correlated with a poorer outcome. No significant difference was seen whether three or six segments of GLS were used. The data were similar to previous studies that demonstrated that a SRV GLS < −10% was associated with poorer outcome [20], while a GLS lower than −13.3% identifies a higher risk of MACEs. 

The presence of moderate to severe or severe tricuspid regurgitation, previously described as associated with a higher incidence of adverse events, especially in ccTGA patients [5], was an important predictor of outcome: HR 3.2 95%CI 1.2–9.1, *p* = 0.02. The current guidelines suggest tricuspid valve replacement for severe TR in symptomatic adults with preserved or mildly depressed systemic ventricular function (ccTGA) and in AS patients without significant ventricular systolic dysfunction (EF > 40%) regardless of symptoms [21,22]. 

Exercise tolerance should be assessed every 24–36 months according to the clinical status; evidence of worsening exercise capacity with reveal of cardiogenic limitation such as lower O2 pulse and higher VE/VCO2 corroborated by the clinical condition could indicate an invasive evaluation with cardiac catheterization. Advanced imaging evaluation should be regularly performed every 3–5 years in stable patients and eventually every other year or less in patients with complications. 

Exercise capacity in patients with SRV is reduced, with a peak VO2 of 24.5 ± 9.4 mL/kg/min (67 ± 20% of the predicted value), similar to previously published data: VO2 27.14 ± 4.3 mL/kg/min [23]; VO2 28.6 ± 8.3 mL/kg/min for AS patients and 25.6 ± 7.1 mL/kg/min for ccTGA patients [8]; 21.5 ± 5.8 mL/kg/min (57 ± 14% of the predicted value) [24]; 23.3 ± 6.9 mL/kg/min (68 ± 16.6% of the predicted value) [25]; and 27 ± 7 mL/kg/min [26]. Chronotropic incompetence, frequent in this category of patients [24], was associated with a poorer outcome [8].

Pregnancy is generally at high risk in this cohort of patients (class mWHO III with an increased risk of maternal mortality or severe morbidity, estimated around 19–27%) [27]. Therefore, pregnancy should be discussed and supported by a multidisciplinary ACHD team involving cardiologists, gynecologists, obstetricians, cardiac surgeons, radiologists, and psychologists. An interesting component of the data in our study is the high number of pregnancies that were relatively well tolerated with no maternal deaths. A case of vertical transmission of ccTGA was evidenced; further multidisciplinary studies could be useful to better understand the risk of transmission of these complex CHD cases and eventually the genes involved. [28,29,30] Given the risk of an irreversible decline in RV function during pregnancy, we suggest exhaustive multidisciplinary counseling for fertile women seeking pregnancy [27].

ACHD patients experience a chronic disease; therefore, the quality-of-life assessment is important in this cohort of patients; every therapeutical change should evaluate both the clinical and the QoL benefits. For disease related-QoL evaluation in patients with acquired heart diseases, different questionnaires are available, such as the Minnesota Living with Heart Failure Questionnaire (MLHFQ) or the Kansas City Cardiomyopathy Questionnaire (KCCQ). The QoL evaluation in ACHD with SRV does not have a standard questionnaire, and we chose for our study a well-known generic instrument, the SF-36, because it has already been used in ACHD patients, and it has been validated for the Italian population. Contrary to expectations, the health-related quality of life of these patients was similar to the QoL of the Italian population. This was generally explained for the ACHD population by a stronger sense of coherence [31,32].

## 5. Conclusions

Long-term follow-up of patients with a systemic right ventricle is characterized by a high incidence of clinical events, prevalently arrhythmias and heart failure, which cause most of the unscheduled hospitalizations. Lower exercise capacity, dilated and dysfunctional SRV, lower RV LS, a higher degree of TR, and the presence of fibrosis were associated with a higher incidence of adverse events. Pregnancy was feasible but associated with a high risk of adverse events; therefore, multidisciplinary counselling should be organized for young women seeking motherhood. The quality of life of patients with SRV was similar to the general Italian population, which might be explained by a stronger sense of coherence associated with the chronical illness.

## Figures and Tables

**Table 1:** Echocardiography results. Echocardiography was performed using the European Association of Cardiovascular Imaging/American Society of Echocardiography guidelines and recommendations. (RV) right ventricle, (FAC) fractional area change, (TAPSE) tricuspid annular plane systolic excursion, (S wave TDI RV) pulsed wave tissue doppler imaging, (RV D1, D2, D3) basal, mid, and longitudinal right ventricle diameter, (RV LS) right ventricle longitudinal strain, (LV EDV) left ventricle end diastolic volume, (LVEF) left ventricle ejection fraction.

	ALL (73)	AS (34)	CCTGA (39)	*p*
RV end diastolic area, cm^2^ (mean ± SD)	34.8 ± 11.1	39.5 ± 10.5	30.6 ± 10.0	<0.001
RV end systolic area, cm^2^ (mean ± SD)	22.2 ± 9	25.8 ± 8.4	19.3 ± 8.6	0.002
RV end diastolic area, cm^2^/m^2^ (mean ± SD)	20.1 ± 5.3	21.6 ± 5.6	18.7 ± 4.6	0.01
RV end systolic area, cm^2^/m^2^ (mean ± SD)	12.9 ± 4.4	14.1 ± 4.3	11.8 ± 4.3	0.03
FAC, % (mean ± SD)	36.8 ± 10.3	36.2 ± 4.3	37.4 ± 11.8	0.65
TAPSE, mm (mean ± SD)	15.3 ± 3.8	14.2 ± 3.8	16.4 ± 3.6	0.02
S wave, TDI RV (mean ± SD)	8.7 ± 2.3	8.4 ± 2.1	9.1 ± 2.3	0.2
RV D1 (mean ± SD)	53 ± 10	57 ± 10	50 ± 10	0.01
RV D2 (mean ± SD)	52 ± 12	55 ± 12	50 ± 12	0.14
RV D3 (mean ± SD)	79 ± 16	86 ± 13	73 ± 15	<0.001
Free wall RV LS (mean ± SD)	−12.1 ± −5.1	−10.2 ± −3.6	−14.1 ± 15.8	0.002
6 segments RV LS (mean ± SD)	−10.9 ± −4.5	−9.3 ± −3.4	−12.4 ± −4.8	0.006
RV volume, strain mL	97 ± 52	104 ± 57	89 ± 45	0.25
Tricuspid regurgitation, (n, %) 1 2 3 4	24 (32.8) 17 (23.2) 13 (17.8) 6 (8.2)	12 (35.2) 9 (28.1) 7 (21.8) 4 (12.5)	12 (30.7) 8 (20.5) 6 (18.7) 2 (5.1)	0.2
Tricuspid prosthesis	10 (13.6)	2 (6.2)	8 (20.5)	<0.001
Left atrial volume, mL/m^2^ (mean ± SD)	39 ± 25	38 ± 23	40 ± 26	0.65
Right atrial volume, mL/m^2^ (mean ± SD)	21 ± 13	18 ± 11	24 ± 13	0.06
LV EDV mL (mean ± SD)	56 ± 28	57 ± 29	55 ± 27	0.74
LVEF %, (mean ± SD)	63 ± 10	65 ± 9	61 ±10	0.10

**Table 2:** CMR results. CMR was performed using a 1.5-Tesla Magnetic Resonance Unit with a standardized protocol for the evaluation of the systemic right ventricle. (EDV RV) end diastolic volume right ventricle, (ESV RV) end systolic volume right ventricle, (RV EF) right ventricle ejection fraction, (EDV LV) end diastolic volume left ventricle; (EDS LV) end systolic volume left ventricle, (LV EF) left ventricle ejection fraction, (RV mass) right ventricle mass, (LGE) late gadolinium enhancement.

CMR	ALL (52)	AS (28)	CCTGA (24)	*p*
EDV RV, mL/m^2^ (mean ± SD)	117 ± 40	127 ± 46	106 ±30	0.07
ESV RV, mL/m^2^ (mean ± SD)	62 ± 34	72 ± 39	50 ± 24	0.02
RV EF, % (mean ± SD)	49 ± 12	47 ± 11	53 ± 12	0.06
EDV LV, mL/m^2^ (mean ± SD)	76 ± 27	74 ± 26	78 ± 29	0.56
ESV LV, mL/m^2^ (mean ± SD)	32 ± 19	30 ± 16	34 ± 23	0.43
LV EF, % (mean ± SD)	60 ± 12	61 ± 9	59 ± 15	0.53
RV Mass, g/m^2^ (mean ± SD)	41 ± 17	40 ± 20	43 ± 14	0.55
LGE, % (n, %)	22 (42.3)	14 (50)	8 (33.3)	0.14

**Table 3:** CPET results. Cardiopulmonary exercise test (CPET) was performed on an upright cycle ergometer using a continuous ramp protocol until muscular exhaustion with continuous monitoring of expiratory gas. (SBP) systolic blood pressure, (Peak VO2) peak oxygen uptake, (HR) heart rate, (RER) respiratory exchange ratio, (VAT) ventilatory anaerobic threshold, (VE/VCO2) slope relationship between minute ventilation and carbon dioxide production, O2 pulse is VO2/heart rate (ml of oxygen consumed per heartbeat). If there is no desaturation during exercise, then O2 pulse can be an indicator of stroke volume.

CPET	OVERALL (57)	AS (34)	CCTGA (39)	*p*
Baseline HR, mean ± SD	70 ± 15	65 ± 16	75 ± 14	0.02
Chronotropic index, mean ± SD	0.74 ± 0.21	0.72 ± 0.24	0.78 ± 0.17	0.5
Peak SBP, mean ± SD	143 ± 23	144 ± 23	140 ± 23	0.49
Watts, mean ± SD	122 ± 41	122 ±38	121 ± 45	0.93
Peak VO2 (mL/kg/min), mean ± SD	24.5 ± 9.4	22.2 ± 7.2	27.2 ± 11	0.04
Percentage predicted VO2, mean ± SD	67 ± 20	61 ± 16	75 ± 21	0.007
Peak HR, mean ± SD	148 ± 25	142 ± 38	152 ± 26	0.25
RER, mean ± SD	1.2 ± 0.1	1.2 ± 0.09	1.2 ± 0.1	0.6
VAT (mL/kg/min), mean ± SD	17.7 ± 6.8	15.7 ± 4.8	20.3 ± 8.1	0.02
VE/VCO2 slope, mean ± SD	34 ± 8	37 ± 8	31 ± 6	0.02
O2 pulse (% predicted) mean ± SD	87 ± 27	76 ± 24	101 ± 23	0.001

**Table 4:** Univariate analysis. (NYHA Class) The New York Heart Association Classification, (RV) right ventricle, (FAC) fractional area change, (TAPSE )tricuspid annular plane systolic excursion, (S wave TDI RV) pulsed wave tissue doppler imaging, (TR > 2) tricuspid valve regurgitation more than moderate; (RV D) right ventricle diameter, (RV LS) right ventricle longitudinal strain, QRS complex at ECG; (Peak VO2) peak oxygen uptake, (EDV RV) end diastolic volume right ventricle, (ESV RV) end systolic volume right ventricle, (RV EF) right ventricle ejection fraction, (LGE) late gadolinium enhancement.

	HR	95% CI	*p*
NYHA class	2.8	1.8–4.7	<0.0001
RV end diastolic area (cm^2^)	1.1	1.0–1.2	0.07
RV end systolic area (cm^2^)	1.1	1.0–1.2	0.02
FAC (%)	0.9	0.8–0.96	<0.001
TAPSE (cm)	0.86	0.7–0.99	0.01
S wave, TDI RV (cm/s)	0.7	0.6–0.9	0.01
TR > 2	3.2	1.2–9.1	0.02
RV DI (cm)	1.7	1.1–2.7	0.001
RV DII (cm)	1.9	1.3–2.7	0.001
Free wall RV LS	0.89	0.8–0.98	0.01
6 segments RV LS	0.87	0.78–0.98	0.01
Left atrium volume mL/m^2^	1.03	1.01–1.05	<0.001
Right atrium volume mL/m^2^	1.03	1.0–1.06	0.008
QRS duration (ms)	1.04	1.02–1.06	<0.001
Percentage predicted VO2	0.96	0.93–0.99	0.05
EDV RV, mL/m^2^	1.02	1.01–1.03	<0.001
ESV RV, mL/m^2^	1.02	1.01–1.03	<0.001
RV EF, %	0.9	0.8–0.99	0.008
LGE	12	2.6–59	0.009

## Data Availability

Statements are available in section “MDPI Research Data Policies” at https://www.mdpi.com/ethics.
